# Cannabis Use and Subjective Cognitive Decline Among Middle-Aged and Older Adults

**DOI:** 10.1093/geroni/igab046.706

**Published:** 2021-12-17

**Authors:** Maritza Dowling

**Affiliations:** George Washington University - School of Nursing, Washington, District of Columbia, United States

## Abstract

Cannabis is the most commonly-used drug in the US, with older adults being the fastest-growing group of users. National surveys among 50+ adults found poorer executive function among current or past cannabis users, but better performance with daily users. However, there is little evidence linking levels of cannabis use and subjective cognitive decline (SCD) among middle-age and older adults. This study sought to examine the association between levels of cannabis use in the past 30 days and SCD during the past 12 months using Behavioral Risk Factor Surveillance System (BRFSS) data (2016-2019) from 45+ individuals while controlling for demographics, chronic conditions, exercise, general and mental health. Logit models with SCD as outcome were estimated using complex survey weights. Multiple group analyses examined differences across age groups: 45-64 vs. 65+. Levels of cannabis use in the past 30 days were categorized as: no use, 1-4 days, 5-20 days; and 21-30 days. Adjusted results indicated that those who reported no cannabis use were from 35% to 47% less likely to report SCD compared to cannabis users at all levels: 1-4 days (OR=0.65, 95%CI=0.46, 0.92); 5-20 days (OR=0.53, 95%CI=0.36,0.78); 21-30 days (OR=.61, 95%CI=0.45,0.83). In multi-group analyses, levels of cannabis use effects on SCD remained statistically significant in the 45-64 age group, but not in the 65+ group. Further research targeting SCD is needed to design interventions particularly for middle-age cannabis users whose health has been compromised by disease or age-related vulnerabilities and are at greater risk for adverse cognitive outcomes from cannabis use.

